# Haplotype-resolved genome assembly and implementation of VitExpress, an open interactive transcriptomic platform for grapevine

**DOI:** 10.1073/pnas.2403750121

**Published:** 2024-05-28

**Authors:** Anis Djari, Guillaume Madignier, Olivia Di Valentin, Thibault Gillet, Pierre Frasse, Amel Djouhri, Guojian Hu, Sebastien Julliard, Mingchun Liu, Yang Zhang, Farid Regad, Julien Pirrello, Elie Maza, Mondher Bouzayen

**Affiliations:** ^a^Laboratoire de Recherche en Sciences Végétales–Génomique et Biotechnologie des Fruits-UMR5546, Université de Toulouse, CNRS, Université Paul Sabatier, Institut Polytechnique de Toulouse, Auzeville Tolosan 31326, France; ^b^Fondation Jean Poupelain, Cognac, Javrezac 16100, France; ^c^Conservatoire du vignoble charentais, Institut de Formation de Richemont, Cherves-Richemont 16370, France; ^d^Key Laboratory of Bio-Resource and Eco-Environment of Ministry of Education, College of Life Sciences, Sichuan University, Chengdu 610065, China

**Keywords:** *Vitis vinifera*, haplotype-resolved genome assembly, transcriptomic platform

## Abstract

Haplotype-resolved genome assemblies were produced for Chasselas and Ugni Blanc, two heterozygous *Vitis vinifera* cultivars by combining high-fidelity long-read sequencing and high‐throughput chromosome conformation capture (Hi-C). The telomere-to-telomere full coverage of the chromosomes allowed us to assemble separately the two haplo-genomes of both cultivars and revealed structural variations between the two haplotypes of a given cultivar. The deletions/insertions, inversions, translocations, and duplications provide insight into the evolutionary history and parental relationship among grape varieties. Integration of de novo single long-read sequencing of full-length transcript isoforms (Iso-Seq) yielded a highly improved genome annotation. Given its higher contiguity, and the robustness of the IsoSeq-based annotation, the Chasselas assembly meets the standard to become the annotated reference genome for *V. vinifera*. Building on these resources, we developed VitExpress, an open interactive transcriptomic platform, that provides a genome browser and integrated web tools for expression profiling, and a set of statistical tools (StatTools) for the identification of highly correlated genes. Implementation of the correlation finder tool for *MybA1*, a major regulator of the anthocyanin pathway, identified candidate genes associated with anthocyanin metabolism, whose expression patterns were experimentally validated as discriminating between black and white grapes. These resources and innovative tools for mining genome-related data are anticipated to foster advances in several areas of grapevine research.

## Significance

In contrast to most crops, *Vitis vinifera* benefited little from classical breeding due to their heterozygosis. Strikingly, the main cultivated grape varieties that we see today have remained the same as they were centuries ago; they have been poorly enriched with traits to adapt to changing environment. However, climate change and concerns about environment call for major changes in viticulture that require transitioning to knowledge-based concepts and advanced genomics tools. We report here the generation of haplotype-resolved genome assemblies for two grapevine cultivars and the setup of VitExpress, an open interactive transcriptomic platform, providing genome browser and integrated web tools for expression profiling and gene correlation studies. These community resources and tools are anticipated to foster advances in several areas of grapevine research.

Viticulture is a major agricultural activity worldwide, and wine production is both economically and culturally important across many cultures. Wine is associated with the celebration of pleasant moments of life and has been associated with religious ceremonies in multiple civilizations for millennia. Strikingly, in contrast to most agricultural crops, the main varieties or “cepages” universally grown today are much the same as those used centuries ago (1). This longevity can be at least partly explained by a historic cultural dimension since different producing regions have built the reputation of their wine on a specific grape variety and do not wish to change it so as not to alter the myth that goes with their story. Nevertheless, climate change and increasing consumer demand for environmentally friendly viticultural practices call for improved grapevine varieties better adapted to current needs (2). One major characteristic of cultivated vines is their heterozygosity that prevents any improvement by genetic crossing that would necessarily lead to loss of the initial variety (3). The advent of modern genomics and technologies opens up unprecedented opportunities to bypass some of the limitations that have hampered the improvement of cultivated grape varieties. Indeed, the development of advanced tools and resources is a prerequisite for any expected progress in our understanding of the processes and factors underpinning important grapevine traits (4). A necessary first step in knowledge-based variety improvement is to have a reference genome sequence of the highest available quality matched to high-throughput transcriptomic tools that enable straightforward identification of genes and regulatory networks controlling trait variation and phenotypic diversity among different grape varieties. As with many other crops, viticulture faces the challenges presented by global warming, and there is ample evidence that climate change is affecting grape and wine production through impacting plant vegetative growth and berry development. The acceleration of phenological phases has been reported in many wine-producing areas, including precocious bud break, early flowering, and fast ripening (5). Also, high sugar accumulation in berries associated with warmer temperatures leads to increased alcohol content and altered taste and aroma (4). Another consequence of climate change is the resurgence of diseases that require more pesticide treatments, which runs counter to the current societal demand for environmentally friendly agriculture (6, 7).

It is noteworthy that classical breeding, based on crosses between selected genotypes aiming to create new cultivars with improved performance, is inappropriate for grapevine since any progeny lose the identity of the parent grape variety and the terroir on which the reputation of the wine is based. In this context, new breeding technologies are anticipated to open opportunities to improve the widely used grape cultivars without necessarily losing their genetic identity (8). These strategies require development of efficient methods for genome editing and organ regeneration not yet available for grape. Either way, the implementation of such strategies requires development of powerful data mining tools enabling the treatment of mass -omics data in order to identify relevant gene target (4). The amount of grape transcriptomic data has continuously increased since the release of the first high-quality grape genome sequence (9), opening the possibility of system biology approaches to target trait improvement. In order to take advantage of these conceptual and methodological shifts, powerful user-friendly data mining tools are needed. For instance, the heterogeneity of data from different sources and technological platforms requires a substantial curation effort to enable efficient comparative visualization of gene expression profiles across publicly available databases. Several existing databases like Gramene.org (10) deal with many plant species, others like grape-RNA (11) are specific to grape. For instance, VESPUCCI (12) grape transcriptomic database provides normalized expression data for microarray and RNA-seq datasets using command line. However, this database needs to be queried programmatically using the GraphQL interface, Python, or R packages, making it difficult to use by scientists lacking sufficient computing skills.

Grape is a diploid plant with a heterozygous genome and the current reference genome sequence consists of an artificial construction of a nearly homozygous genome that does not reflect the complexity of heterozygous cultivars. Despite the overall very good quality of the PN40024.v4 (13) assembly (PNv4), there are challenges associated with the inherent limitations of short-read DNA sequencing technologies. Furthermore, heterozygosity, high levels of repeat sequences and the lack of parental information make a haplotype-resolved assembly difficult to achieve. The advent of long-read sequencing technologies opens a broad avenue for de novo sequencing and assembly of heterozygous genomes, although it remains a challenging task when the information about the parental lines is missing (14–17). While a telomere-to-telomere assembly has been produced recently using long-read sequencing strategies (18), it did not provide a haplotype-resolved version because it used the nearly homozygous PN40024 cultivar. More recently, a haplotype-resolved genome assembly has been reported for the red flesh “teinturier” cultivar Yan73 (19), however, without providing a de novo gene annotation of their assembly. We report here on the implementation of combined long-read high-fidelity PacBio sequencing and Hi-C technologies to produce complete haplotype-resolved genome assemblies for both Chasselas and Ugni Blanc, two widely cultivated grape varieties. Chasselas is interesting as its early maturity serves as reference for the maturation stages of grape (20), and Ugni Blanc is the major variety of the Cognac-producing area in addition of being used worldwide for white wine production (21). Overall, our study allowed the generation of an upgraded reference genome sequence for *Vitis vinifera* and set the foundation for building VitExpress, an open interactive transcriptomic platform that provides versatile tools for data mining and the identification of genes of interest.

## Results and Discussion

### Haplotype-Resolved Genome Assembly.

Cultivated grapevines are genetically heterozygous plants, and in this regard, a major advantage of a pipeline integrating HiFi long reads and contact map data generated by Hi-C sequencing is to allow producing, in an unprecedented way for *V. vinifera*, haplotype-resolved assemblies of the parental genomes for any grape cultivar with unavailable parental data. To this end, we established a de novo assembly and annotation pipeline of the grape genome that comprises three main steps: A) raw data production, profiling and validation, B) assembly of the circular consensus sequences (CCS) and their integration with the contact map data produced by Hi-C, and C) structural and functional annotation based on the use of isoform sequencing (PacBio Iso-Seq) RNA data (*SI Appendix*, Fig. S1). We implemented this pipeline for Chasselas and Ugni Blanc grape cultivars, two varieties cultivated worldwide. For Chasselas, initially served as *V. vinifera* representative in the species classification table (22), the PacBio HiFi technology produced 25.5 Gb CCS providing 51× coverage of the grape genome with an average of 18 kb read length. Approximately equivalent metrics were obtained for Ugni Blanc with 26 Gb CCS, 52× coverage, and 17 kb average read length. Genome scope profiling estimated the genome sizes of Chasselas and Ugni Blanc to be 492 Mb and 497 Mb, respectively (*SI Appendix*, Fig. S2), in line with the estimated 500 Mb genome size for *V. vinifera* (9). K-mer genome profiling shows complex models with 25× and 50× coverage peaks standing for heterozygous and homozygous sequences, respectively, consistent with diploid genomes for both cultivars (*SI Appendix*, Fig. S2). The two genomes show high levels of heterozygosity (1.4% for Chasselas and 1.52% for Ugni Blanc) due to their untouched real-field genetic pool, in contrast to the partially homozygous Pinot Noir genome previously used as a reference for grapevine.

**Fig. 1.**
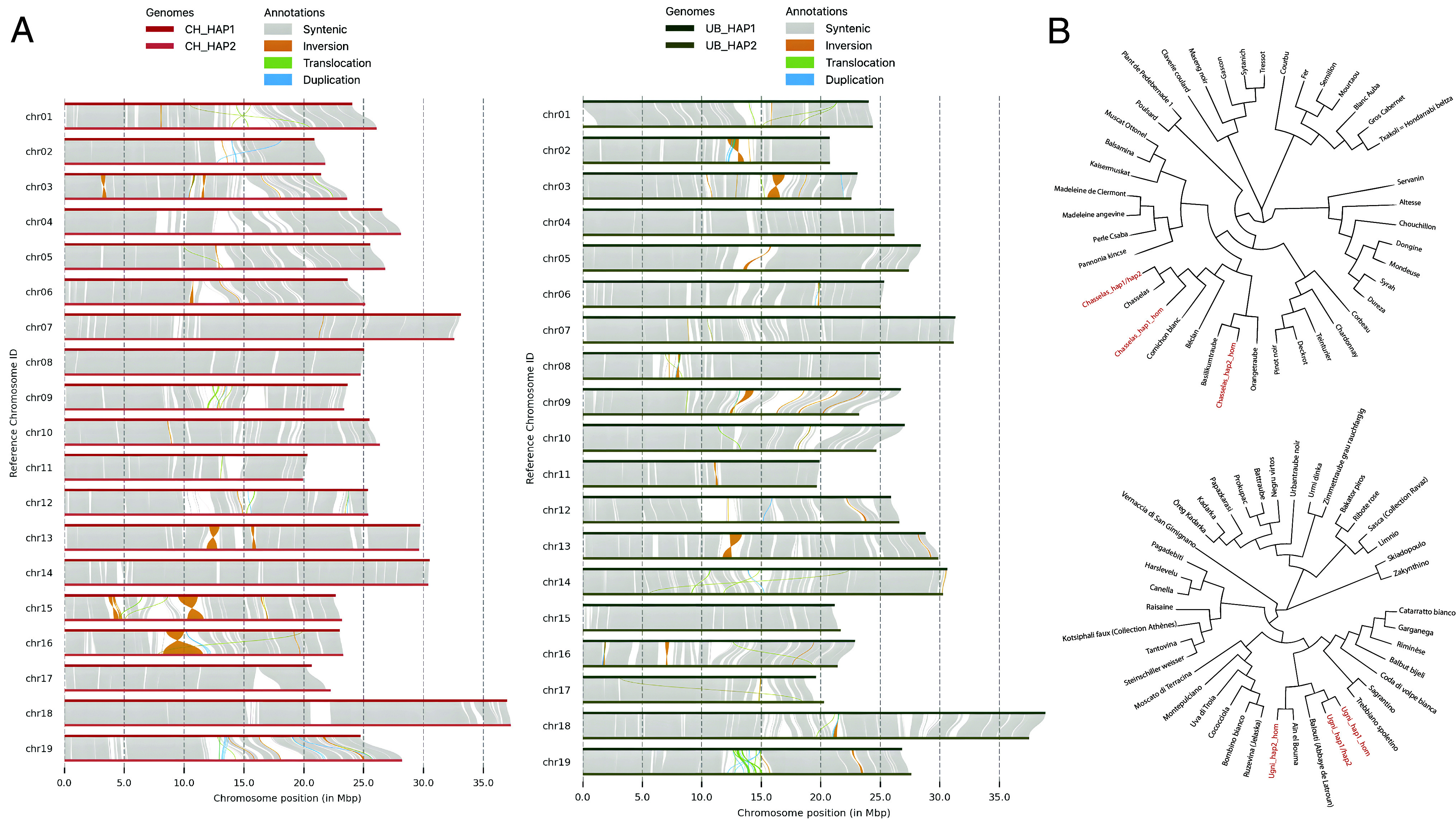
Structural variations between haplotypes and phylogenetic relationships. (*A*) Synteny and rearrangement plot between the chromosomes of the haplotypes of Chasselas (*Left*) and Ugni Blanc (*Right*). Haplotype 1 at the *Top* and haplotype 2 at the *Bottom*. (*B*) SNP-based unrooted phylogenetic tree of the 40 closest cultivars to Chasselas (*Top*) and Ugni Blanc (*Bottom*), built using the neighbor-joining tree. The data used to build the phylogenetic trees were retrieved from the GrapeReSeq 20k Vitis genotyping chip and processed as described by ref. 23. The original data are available at https://urgi.versailles.inra.fr/Species/Vitis/GrapeReSeq_Illumina_20K.

The genome assemblies were subjected to two levels of structural and comparative assessments. Following Hi-C manual curation the total numbers of contigs used for the generation of Chasselas and Ugni Blanc assemblies, 57 and 63 respectively, are lower than those for the PN_T2T and PNv4 assemblies which required 189 and 2,646 contigs, respectively (Table 1). Each of the 19 chromosomes of Chasselas and PN_T2T is made of a single contig, the remaining contigs are unplaced and pooled in the virtual chromosome 0 (Chr00). As a matter of improvement, Chr00 in Chasselas is made of 38 contigs (3.8 Mb) while it contains 170 (9.7 Mb) and 556 (12.5 Mb) contigs in PN_T2T and PNv4, respectively. The 19 largest contigs in Chasselas and PN_T2T form the 19 chromosomes while 2,090 contigs are required for the construction of PNv4 chromosomes, which clearly emphasizes the power of the long-read sequencing technology (Table 1). Our assembly pipeline generated highly contiguous chromosome-scale pseudomolecules of the Chasselas haplotypes made of 33 and 29 contigs, whereas for Ugni Blanc 56 and 65 contigs are needed (Table 2). Remarkably, 12 chromosomes are covered with a single contig in Chasselas haplotypes, while for Ugni Blanc haplotypes all chromosomes are covered with 5 contigs at most, except chromosome 12 that is constructed with 9 contigs (Table 2). Comparison of the haplo-genomes within a given cultivar reveals structural variations between the two haplotypes including multiple deletions/insertions, inversions, translocations, and duplications (Fig. 1). Within the Chasselas cultivar, chromosomes 7, 8, and 18 exhibit high structural conservation, whereas chromosomes 3, 13, 15, and 16 display several structural rearrangements (Fig. 1*A*). A similar picture emerges for Ugni Blanc with some chromosomes exhibiting higher level of rearrangements (Fig. 1*A*). The robustness of haplotype-resolved assemblies was further validated by in silico genotyping based on SNP marker alleles (23) showing that Chasselas-hap1 associates primarily with Cornichon Blanc and Beclan in the phylogenetic tree (Fig. 1*B*). Consistently, Cornichon blanc was obtained by genetic cross between Chasselas and Bicane, according to the Vitis International Variety Catalogue (VIVC database) (24). As for Chasselas-hap2, the closest cultivars are Basilicum Traube and Orange Traube, both obtained by crosses between Chasselas and Pinot Noir (VIVC database). Interestingly, Cornichon Blanc, Basilicum Traube, and Orange Traube cultivars that have Chasselas as one of the parental ancestors show close relationship with Chasselas-hap1 and hap2. The same genotyping procedure indicated that the Ugni Blanc-hap1 genome is most closely related to Baluti, a cross between Trebbiano and Granacha Tinta (25), while Ugni Blanc-hap2 is closely related to Ain Elbouma (25), a cross between Trebbiano and Olivette Blanche (Fig. 1*B*). Considering that Trebbiano is an alternative name for Ugni Blanc (24), the genotyping indicates that the closest cultivars of Ugni Blanc-hap1 and hap2 are those having Ugni Blanc as a parental ancestor. The phylogenetic analysis provides clues to the evolutionary history and genetic relatedness of the grape varieties studied, giving insight into their shared ancestry and divergence while confirming the position of Chasselas and Ugni Blanc among other *V. vinifera* cultivars. Altogether, the data emphasize the added value of haplotype-resolved assemblies in revealing the parental relationship among grapevine cultivars. This information also provides a means to investigate the ancestry and descent relationships between different cultivars and hence trace the origins of important traits.

**Table 1.** Assembly metrics and BUSCO genome score for each primary assembly

		CH_REF	UB_REF	PN_T2T	PNv4
Contigs	Number	57	62	189	2,646
	N50 contig length	25.4 Mb	19 Mb	26.8 Mb	445 Kb
	L50 contig count	9	10	9	321
Scaffolds					
	Total size of scaffolds^*^	499.9 (496.1) Mb	494.6 (491) Mb	504.6 (494.9) Mb	474.7 (462.2) Mb
	Longest scaffold	37.9 Mb	38.8 Mb	36.7 Mb	34.9 Mb
	Median scaffold	25.1 Mb	25.7 Mb	25.9 Mb	24.2 Mb
	N50 scaffold Length	25.4 Mb	26 Mb	26.8 Mb	24.3 Mb
	L50 scaffold count	9	9	9	25
	GC%	35.1	35.1	35.3	34.5
Chromosomes^†^					
	Telomeres	35	35	36	27
	Contigs in main chromosomes^‡^	19	33	19	2,090
	Contigs in chromosome 00	38	29	170	556
	% of assembly in chromosomes	99.3%	99.3%	98.1%	97.3%
	% of assembly in chromosome 00	0.7% (3.8 Mb)	0.7% (3.6 Mb)	1.9% (9.7 Mb)	2.6% (12.5 Mb)
Assembly completeness	Complete BUSCOs (C)^§^	98.5%	98.5%	98.3%	98.4%
	C & single-copy	97.0%	97.0%	96.6%	96.2%
	C & duplicated	1.5%	1.5%	1.7%	2.2%
	Fragmented	0.9%	0.9%	1.1%	1.1%
	Missing	0.6%	0.6%	0.6%	0.5%

^*^The number in brackets relates to the total size without chr00.

^†^The 20 chromosomes are made of 20 scaffolds consisting in the 19 main chromosomes and a virtual chromosome 00.

^‡^Number of contigs used to build the 19 main chromosomes.

^§^BUSCO genome.

**Table 2.** Contigs per chromosome for each haplotype in Chasselas and Ugni Blanc genome assemblies

Chromosomes																				
	01	02	03	04	05	06	07	08	09	10	11	12	13	14	15	16	17	18	19	Total
CH_HAP1	2	1	2	1	1	1	1	1	1	1	1	2	2	7	1	1	4	2	1	33
CH_HAP2	3	1	3	1	1	1	1	1	1	1	2	2	1	1	1	1	2	2	3	29
UB_HAP1	3	5	5	2	2	1	2	5	3	3	3	4	5	2	2	5	1	1	2	56
UB_HAP2	1	4	3	2	2	5	4	3	4	2	5	9	3	4	1	3	5	2	3	65

### Producing an Advanced Reference Genome Sequence for *V. vinifera*.

The combination of HiFi and Hi-C technologies yielded 35 out of the 38 telomeres giving rise to telomere-to-telomere (T2T) single contigs for most chromosomes (Table 1 and *SI Appendix*, Fig. S3*B*). The 19 chromosomes are covered with 19 and 33 contigs in Chasselas and Ugni Blanc assemblies, respectively (Table 3). Given the higher contiguity of the Chasselas assembly over the Ugni Blanc as confirmed by the Hi-C data (*SI Appendix*, Fig. S3*A*), this assembly was therefore retained for subsequent annotation and overall suitability to be used for building a higher quality reference genome sequence for *V. vinifera*. The size of the Chasselas primary assembly (496.1 Mb) excluding chr00 (Table 1) is comparable to that of PN_T2T (494.9 Mb) while PNv4 assembly is 34 Mb smaller, the missing part is equivalent to the size of one of the largest grape chromosomes (18, 26). This is consistent with the higher contig number (556) placed in PNv4 chr00 compared to 170 and 38 contigs in PN_T2T and Chasselas, respectively (Table 1). Moreover, qualitative assessment performed using 1,614 universal orthologous conserved genes from Embryophyta.odb10, revealed a higher BUSCO (27) score (98.5%) compared to PN_T2T (98.3%) with the single copy genes reaching 97% in Chasselas vs. 96.6% in PN_T2T (Table 1). This reflects the higher proportion of duplicated and fragmented genes in PN_T2T assembly.

**Table 3.** Contigs per chromosome for Chasselas, Ugni Blanc, PNv4, and PN_T2T genome assemblies

Chromosomes																				
	01	02	03	04	05	06	07	08	09	10	11	12	13	14	15	16	17	18	19	00
CH_REF	1	1	1	1	1	1	1	1	1	1	1	1	1	1	1	1	1	1	1	38
UB_REF	2	2	2	1	2	2	1	2	1	2	3	3	2	2	1	1	1	1	2	29
PNv4	84	81	108	89	111	87	207	81	136	161	89	90	131	116	88	116	63	137	119	556
PN_T2T	1	1	1	1	1	1	1	1	1	1	1	1	1	1	1	1	1	1	1	170

**Fig. 2.**
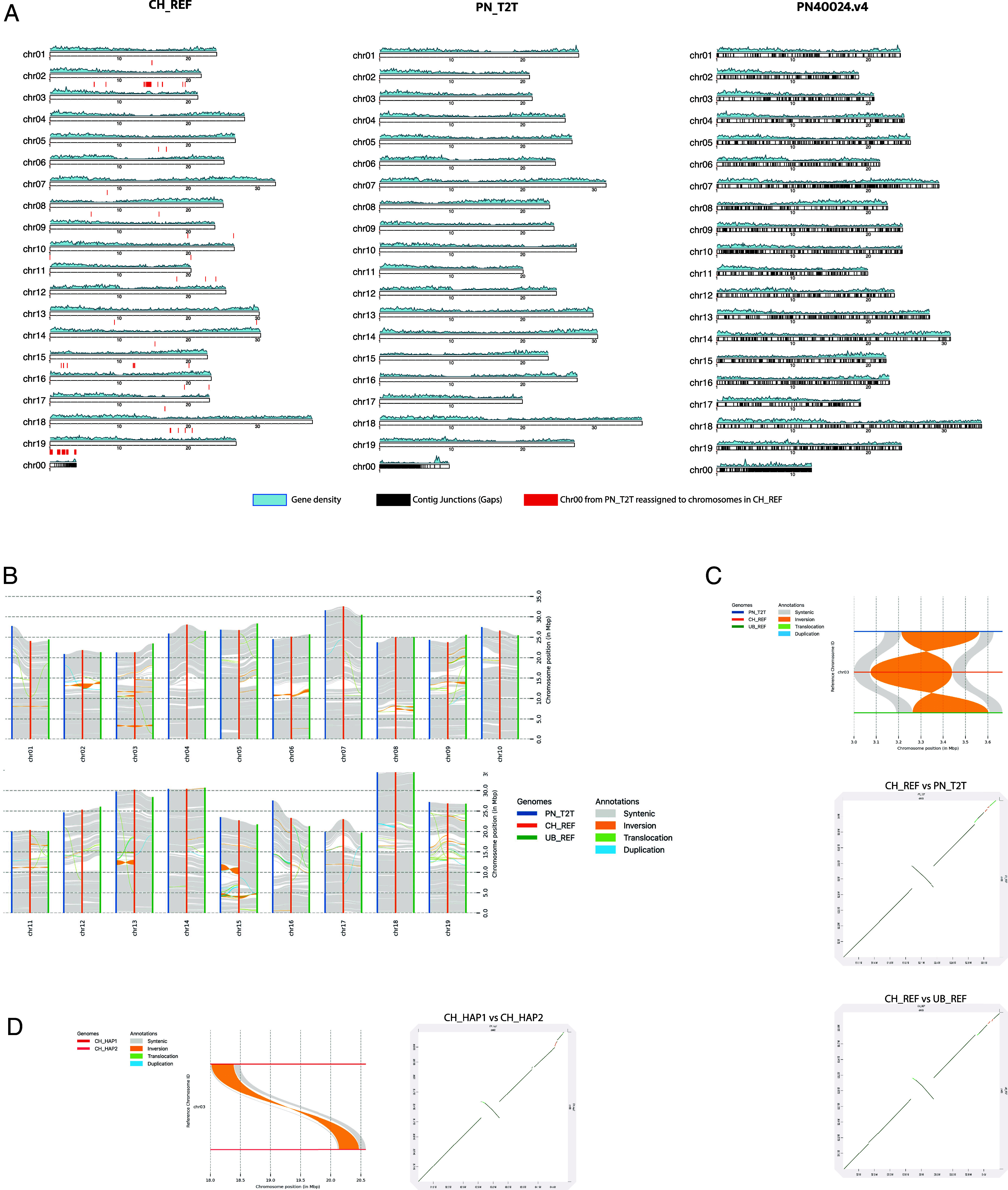
Comparative assessment of primary genome assemblies. (*A*) Karyoplot ideogram of Chasselas (*Left*), Pinot Noir PN_T2T (*Middle*) and PN40024.V4 (*Right*) chromosomes showing gene density (blue). Gaps (black segments) reassignment of PN_T2T’s chr00 fragments are displayed with red line on the Chasselas Ideogram. (*B*) Synteny plot with SyRI showing the syntenic path, structural rearrangements, and unaligned regions between PN_T2T (blue) Chasselas (orange) and Ugni Blanc (green). (*C*) Inversion event observed on the Chasselas chromosome 3 compared to PN_T2T and Ugni Blanc (*Top*). Dotplot of the inversion for Chasselas vs. PN_T2T (*Middle*) and for Chasselas vs. Ugni Blanc (*Bottom*). (*D*) Focus on the inversion observed on chromosome 3 of the Chasselas assembly with synteny plot and dotplot. This inversion is present in only one of Chasselas haplotypes.

As shown by the dot plot analysis (*SI Appendix*, Fig. S4*A*), the chromosome-scale comparison reveals the high matching level of the de novo Chasselas genome assembly with the PN_T2T, PNv4, and the Ugni Blanc assemblies. Alignments of assembled scaffolds show a good reconstruction of the 19 chromosomes of the Chasselas and PN_T2T assemblies compared to the highly fragmented PNv4 assembly (Fig. 2*A*). Notably, Chasselas assembly allowed the reassignment of large fragments of PN_T2T virtual chr00 to the appropriate chromosome location, thus highlighting the improvements brought by our de novo assembly (Fig. 2*A*). Chromosomes 3, 16, and 19 benefited the most from these reassignments. Unanchored scaffolds represent 0.7% of the total genome in Chasselas vs. 1.9% and 2.6% in the PN_T2T and PNv4 assemblies, respectively (Table 1). Moreover, synteny studies, performed using Synteny and Rearrangement Identifier software (SyRI) (28), revealed several structural differences between Chasselas, Ugni Blanc, and PN_T2T (Fig. 2*B*). A number of inversion events are observed between PN_T2T and our two assembled genomes. Some of these are cultivar specific likely corresponding to real structural variations, while others might be due to local misassembled or misoriented short contigs during the scaffolding step. For instance, all inversions within PNv4 chromosomes 4, 5, 7, 10, 13, and 18 have been fixed in PN_T2T and Chasselas assemblies confirming the power of long-read sequencing in solving complex genome areas (*SI Appendix*, Fig. S4*B*). However, the large inversion between PN_T2T and Chasselas chr13 likely correspond to a structural variation between the two cultivars, although a misassembly of PN_T2T in this region cannot be ruled out as suggested by the similarity between Chasselas and Ugni Blanc (Fig. 2*B*). Nevertheless, the inversion in chromosome 3 shared by PN_T2T and Ugni Blanc, but not by Chasselas (Fig. 2*C*), suggests that the cultivars showing the same rearrangement event may have common parental ancestors. Interestingly, the inversion on chromosome 3 observed between Chasselas, PN_T2T and Ugni Blanc (Fig. 2*C*) is present solely in hap1 of Chasselas while hap2 bears in this locus the same structure observed in Ugni Blanc and PN_T2T (Fig. 2*D*). Overall, the features revealed by haplotype-resolved assemblies suggest that the three cultivars might have inherited a common chromosome set from parental ancestors. Overall, the synteny studies further stress the benefits of long-read technologies in reducing the occurrence of local misorientations that may potentially lead to erroneous definitions of the environment of implied genes.

### Structural and Functional Annotation.

Prior to the implementation of the annotation procedure, the identification of repeats is mandatory in order to mask the repeat regions and allow higher quality ab initio annotation step. Implementation of the same pipeline for the detection and classification of repeats in Chasselas, Ugni Blanc, and PN_T2T assemblies revealed that 53% of the genome sequence correspond to transposable elements and other repeat elements (*SI Appendix*, Table S1). To minimize the number of false or incomplete genes produced by ab initio approaches for structural gene annotation, we implemented full-length mRNA sequencing to gain higher specificity and deeper characterization of the gene structures. The de novo annotation of the Chasselas genome consisted in a multistep procedure combining mRNA full-length sequencing (IsoSeq) enriched by the PNv4 model and by an evidence-based ab initio approach. To this end, we performed PacBio IsoSeq sequencing of 5 different tissues including root, stem, leaves, flowers, and fruit which allowed to successfully describe 18,172 genes representative of 68,397 related isoforms. The IsoSeq model was then enriched by a “lift over” approach of the PNv4 model using a highly stringent threshold of acceptance of 80% coverage and identity. In addition, de novo structural annotation was also produced by EugeneEp (29) using evidence from multiple plant models. In a final step, the resulting gene model was subjected to validation using the data available for 1,750 RNA-seq samples from public transcriptomic databases. In this way, only protein-coding genes supported by expression signals were retained for the Chasselas annotation model. This multistep annotation model discarded the pseudogenes based on failure to match any hit in nonredundant protein (NCBI) and UniRef 90 (Uniprot) public databases. In total, 32,090 genes were validated as protein-coding genes of which 88% are retrieved in the current list of PNv4 annotated genes (Table 4). It is important to mention that in our subsequent study, we did not use PN_T2T annotation because it corresponds to a lift over of PNv4 annotation which represents the current and most updated reference annotation for the grape genome. Interestingly, our annotation pipeline identified 1,325 novel genes that are not found in the PNv4 annotation (Table 4). Moreover, 904 genes corresponded to a fusion of two or more of the PNv4 annotated genes, and 332 genes in PNv4 are split into two or more genes in our model (Table 4 and *SI Appendix*, Fig. S5). Among the 32,090 genes, functional annotation performed with the BLAST2GO pipeline (30) supported the robustness of the annotation gene model as 24,655 genes are associated with GO terms. The robustness of the annotation is further sustained by the remarkably high BUSCO scores using either Embryophyta (99.7%) or Eudicots (99.5%) datasets (*SI Appendix*, Table S2). Based on Embryophyta and Eudicots datasets, the number of missing genes ranges from 0 to 2 genes, respectively, which is lower than PN_T2T and PNv4 (*SI Appendix*, Table S2). Moreover, the number of duplicated and fragmented genes in Chasselas annotation is far lower than that in the existing PNv4 and PN_T2T annotations (*SI Appendix*, Table S2). It is worth mentioning that the lower BUSCO transcriptome scores of PN_T2T is not surprising because this annotation corresponds to a “lift over” of the PNv4 annotation, and it is therefore limited by the score of the source model. Regarding the naming of the gene identifiers, our annotation adopted the prefix “Vv” as taxonomy ID instead of “Vitvi” to avoid confusion with the current annotation of the PNv4 genome assembly. Considering the higher quality of the Chasselas genome assembly compared to the PNv4 assembly, as substantiated by the better overall metrics, and given the robustness of the IsoSeq-based annotation, the Chasselas genome assembly meets the highest golden standard to become the annotated reference genome for *V. vinifera*.

**Table 4.** Annotation model metrics

	Chasselas		Chasselas vs. PNv4		
	Gene number	Novel genes^*^	Genes covered^†^	Fused genes	Split genes
Chasselas gene model	32,090	1,325	31,128/35,134	904	332

^*^Filtered list containing only those coding for proteins, not overlapping previously described genes. Genes showing premature stop codons (nonsense-mediated decay) or RT-switch were discarded.

^†^80% identity and coverage.

Building on the annotation of the Chasselas genome, we annotated the Ugni Blanc and the four haplo-genomes assemblies by a lift over of the IsoSeq-enriched Chasselas annotation (*SI Appendix*, Table S3). More than 96% of the Chasselas annotated genes were retrieved in Ugni Blanc. As for the haplotypes, 98% and 96% of Chasselas protein-coding genes were lifted to the Chasselas and Ugni Blanc haplo-genomes, respectively (*SI Appendix*, Table S3) which likely reflects the higher genetic proximity of the Chasselas haplo-genomes with the reference Chasselas annotated genome. Combining the synteny and the annotation data identified the genes impacted by structural variance between haplotypes of Chasselas and of Ugni Blanc. Between Chasselas-Hap1 and Hap2, 793 genes are impacted by insertion/deletion events exceeding 1 kb in size, whereas 391 genes are concerned by inversion/translocation events (*SI Appendix*, Table S4). As for the Ugni Blanc genome, 844 genes are concerned by insertion/deletion events and 708 by inversion/translocation when comparing Ugni Blanc-Hap1 and Hap2 (*SI Appendix*, Table S4). Some of the genes impacted by the structural variation represent potential candidate to address their putative involvement in determining the traits specific to each cultivar.

### VitExpress, an Open Web-Based Expression Platform for Mining Grape Transcriptomic Data.

Building on the quality of the reference genome, we constructed VitExpress, an open and versatile interactive transcriptomic database (www.vitexpress.gbfwebtools.fr) gathering a large set of public transcriptomic data selected on the basis of minimum quality requirements. The construction of this transcriptomic platform required many steps including data collection, curation, quantification, normalization, and integration into the VitExpress database (*SI Appendix*, Fig. S6). VitExpress provides a user-friendly web application platform for simplified, yet efficient, data mining. RNA-seq data were downloaded from the NCBI repository and the sample descriptors were manually curated for better integration and removal of inconsistencies. It is important to mention that VitExpress covers a high number of cultivars and conditions that have been curated and thoroughly homogenized which confers to this platform an unprecedented robustness. In total, more than 2,000 samples were processed; of which ~400 were discarded for insufficient sequence quality resulting in low feature assignation. Harmonizing the datasets has been an essential task in order to allow rigorous comparative analysis and ensure data consistency. To this end, we developed for each sample a data processing pipeline from mapping to quantification of the raw sequence data. These raw counts were then normalized to consider parameters such as library size and gene feature length. In total, the VitExpress platform includes 57 projects and 1,673 samples representing 577 conditions manually characterized. These conditions cover six different organs (flowers, berry, shoot, leaf, root, and seed) from 55 different cultivars corresponding to six main developmental processes that cover the entire grapevine lifecycle (germination, leaf development, inflorescence emergence, flowering, fruit development, and berry ripening) (*SI Appendix*, Fig. S7). In addition to *V. vinifera* varieties that represent 87% of the total samples, the VitExpress platform also includes data from other *Vitis* species (*Vitis riparia*, *Vitis sylvestris*, and *Vitis amurensis*) as well as some hybrid varieties (*SI Appendix*, Fig. S7). The overwhelming majority of the data in VitExpress correspond to cultivars used as wine grape (91%) while table grapes account for 7% of the samples.

VitExpress allows visualization and analysis of RNA-seq data through different StatTools (*SI Appendix*, Table S5). The “Expression profiler” tool displays expression patterns through a line chart covering different organs and developmental stages. The “Expression heat map” gives clustered heatmaps of expression patterns for a defined set of genes or conditions. This clustering tool allows searching for both Euclidean distance, using the expression levels, or Pearson distance to perform linear correlation. Based on the expression pattern of a given gene, the “Correlation gene finder” tool displays positively or negatively correlated genes based on a correlation threshold defined by the user. The “Correlation network” tool computes and displays the expression-based weighted correlation network for a selected gene or genes. The “Isoforms browser” displays, for a given gene, RNA-seq signal tracks corresponding to transcript isoforms present in different conditions or tissues.

### VitExpress Allows Categorizing White and Black Varieties Based on Gene Expression Specificity.

**Fig. 3.**
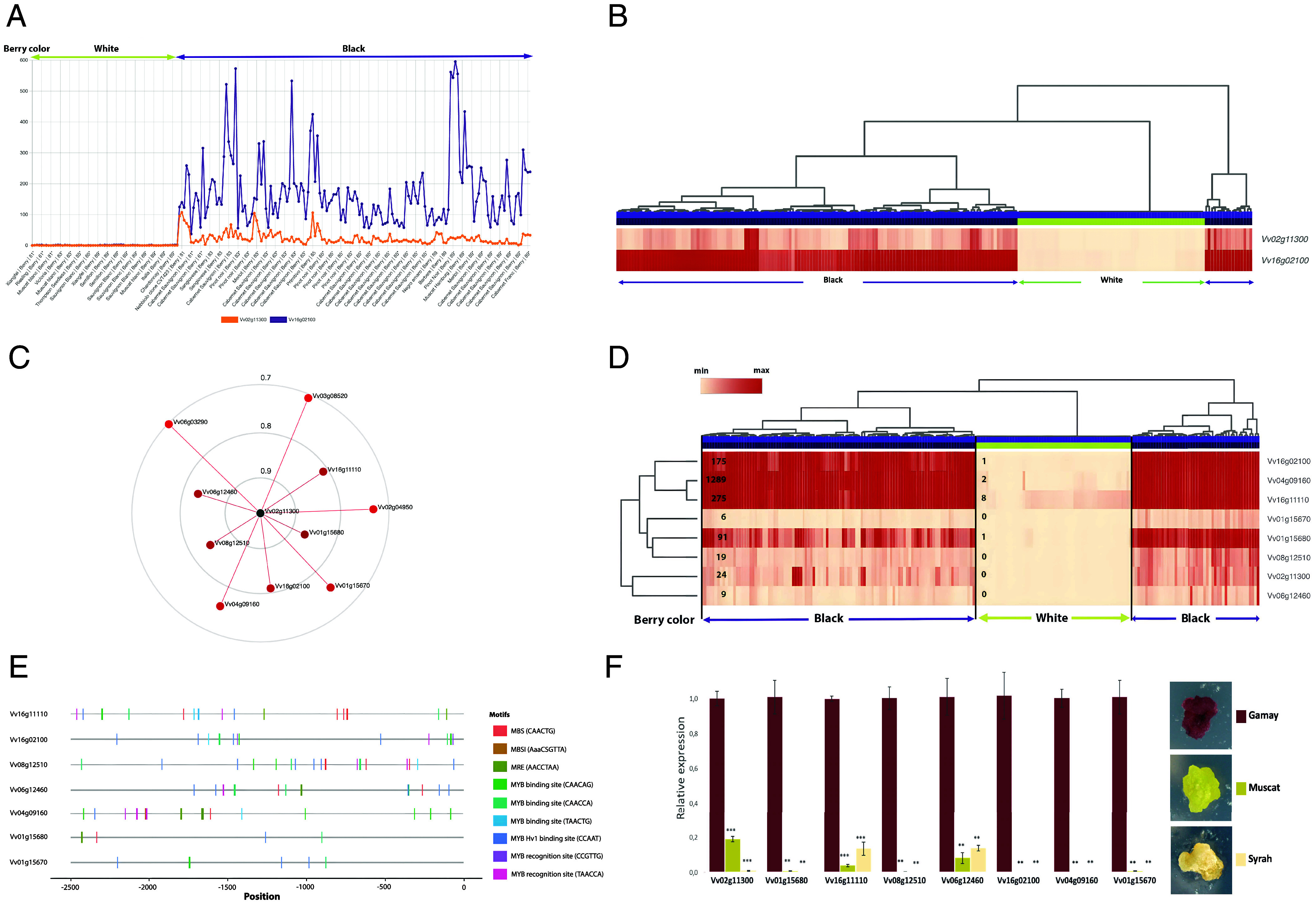
*MybA1*-correlated genes. (*A*) Expression pattern of *MybA1* (Vv02g11300) and *UFGT* (Vv16g02100) as determined by VitExpress using 213 berry samples corresponding to 150 black and 63 white grape berries. (*B*) Heatmap clustering using Pearson correlation of *MybA1* and *UFGT* expression showing their expression in black and white grape varieties. (*C*) Correlation circles displaying the 10 genes whose expression pattern is the most highly correlated with *MybA1* (blue color). The shorter the distance with regard to *MybA1,* the higher is the correlation. (*D*) Heatmap clustering using Pearson correlation between the expression of *MybA1* and the 7 most highly correlated genes (correlation coefficient ≥ 0.75). The average expression level of a given gene in white and black varieties is indicated by the black numbers. (*E*) Putative Myb-binding sites in the promoter region of genes displaying highly correlated expression to *MybA1* as revealed by in silico search using VitExpress. (*F*) Relative expression assessed by qRT-PCR of *MybA1* and the 7 most highly correlated genes in Gamay red callus producing anthocyanin and in white calli, Muscat and Syrah both deficient in anthocyanin pigment biosynthesis. Expression in the white calli is calculated relatively to that of red Gamay callus. *P* value: **P < 0.01 and ***P < 0.001. The *MybA1*-correlated genes are the following: Vv02g11300 *MybA1*; Vv01g15680 Anthocyanin-O-methyltransferase; Vv16g11110 anthoMATE; Vv08g12510 Solute carrier family 35 member F2; Vv06g12460 Flavonoid 3′,5′-hydroxylase2-like; Vv16g02100 UDP-glucose: flavonoid 3-O-glucosyltransferase; Vv04g09160 Glutathione S-transferase; Vv01g15670 Flavonoid 3′,5′-methyltransferase-like; Vv02g04950 Leucoanthocyanidin dioxygenase; Vv03g08520 Serine carboxypeptidase-like 13 and Vv06g03290 Phenylalanine ammonia-lyase.

Pigment accumulation is a major trait for both wine processing and table grapes. Most black grape varieties are characterized by the accumulation of anthocyanin in the skin, whereas berry flesh and vegetative tissues usually lack the ability to produce this pigment. Nevertheless, few grape varieties known as “teinturier” exhibit the ability to produce anthocyanin in flesh tissues of the berry (19). To test the robustness of VitExpress tools and illustrate the new possibilities they offer for the identification of new genes involved in developmental or metabolic processes, we used the anthocyanin metabolism pathway as a case study. The “Expression profiler” tool displayed the expression patterns of the *UFGT* (Vv16g02100) gene, known to mediate a key step in the anthocyanin biosynthesis pathway and that of *MybA1* transcription factor (Vv02g11300), a known regulator of *UFGT* expression (31). Mining the expression data in 213 berry conditions, of which 63 correspond to white grapes and 150 to black varieties, indicated that transcript accumulation of *MybA1* parallels that of *UFGT*, although the absolute levels of UFGT transcripts are always substantially higher than those of its regulator *MybA1* (Fig. 3*A*). Consistent with the role of the two genes in regulating anthocyanin metabolism, the expression of *UFGT* and *MybA1* is detected in black but not in white grape varieties (Fig. 3*B*). We then implemented the “Correlation Gene Finder” tool to search for the 10 most correlated genes whose expression pattern matches that of *MybA1* in 213 conditions of transcriptomic data corresponding to berry samples of black and white grape varieties (Fig. 3*C*). Notably, in addition to the *UFGT* gene, 9 other genes highly correlated with *MybA1* were identified, among which some have not been previously associated with anthocyanin metabolism in grape berries (*SI Appendix*, Table S6 and Fig. 3*C*). Remarkably, the expression of the 7 most highly correlated genes with *MybA1* (Pearson correlation higher than 0.75) allowed the clustering of the grape varieties into two separate blocks corresponding to white and black cultivars (Fig. 3*D*). That is, the white varieties display extremely low or undetectable expression levels for the correlated genes, in contrast to the black varieties that exhibit significant expression levels for the same genes. Four genes (Vv01g15680, Vv16g11110, Vv08g12510, and Vv06g12460) show higher correlation with *MybA1* expression than *UFGT* (*SI Appendix*, Table S6) (32–35). Notably, among the *MybA1*-correlated genes, three (Vv04g09160, Vv02g04950, and Vv01g11110) exhibit higher mean expression levels than *UFGT* in black grapes (Fig. 3*D*). For instance, Vv04g09160 which encodes a putative Glutathione S-transferase, reported to be expressed in Gamay red cell culture, exhibits the highest mean expression level in red grape varieties (33). VitExpress also identified another gene (Vv16g11110) that shows extremely high expression in blackberries and very low expression in white berries. Remarkably, to our knowledge, this gene, which has not been reported previously for its involvement in anthocyanin metabolism, shows high homology with members of the “Antho Multidrug And Toxic compound Extrusion” (anthoMATE) family and is located in the genomic region gathering the anthoMATE genes’ cluster encoding putative vacuolar H+-dependent acylated anthocyanin transporters (36). Consistently, the search for Myb-binding sites in the promoter region of the 7 genes whose expression is highly correlated with *MybA1* revealed the presence of several putative *Cis*-elements (Fig. 3*E* and *SI Appendix*, Table S7) in their promoters as predicted by PlantPAN (37). This clearly suggests that these genes are direct targets of MybA1. Given that the expression of some of these genes is specific to black grape varieties and is highly correlated to *MybA1*, they are likely to play a yet undescribed role in anthocyanin metabolism.

### Experimental Validation of the Expression of *MybA1*-Correlated Genes in Anthocyanin-Producing and Nonproducing Grape Calli.

We took advantage of having available in house in vitro cultivated calli from different grape varieties, all of which are deficient in anthocyanin production except one. Indeed, the Gamay callus displays red pigmentation while all others from different grape types (Fig. 3*F*) exhibit no coloring, regardless of whether they originally produce black or white berries. It is well known that berry tissues cultivated in vitro, either as calli or as liquid cell culture, lose their ability to produce anthocyanin. Interestingly, a soma-clonal variant callus has been isolated in by our group from Gamay with a restored ability to produce anthocyanin red pigment permanently. We then used this unique material to evaluate the expression of genes correlated with *MybA1* in order to experimentally validate the data delivered by the VitExpress platform. RNA extracted from either Gamay red callus or Muscat and Syrah white calli were subjected to qRT-PCR analysis to determine the transcript levels of *MybA1* and its seven most highly correlated genes. The data indicate that MybA1 and UFGT transcripts are expressed at high levels in Gamay red callus, while showing very low or undetectable levels in Muscat and Syrah white calli. Notably, transcript accumulation of the seven *MybA1*-correlated genes remains low in both white calli, which is fully consistent with data obtained by VitExpress using RNA-seq samples corresponding to 63 white and 150 black grape varieties. These experimental data validate those obtained in silico by VitExpress and support the involvement of *MybA1*-correlated genes in the anthocyanin pathway (Fig. 3*F*). Furthermore, the anthoMATE gene (Vv16g11110) identified in our study as associated with production of anthocyanin presents low expression levels in white callus, confirming the power of VitExpress to identify new genes of interest. Consistently, some of these *MybA1*-correlated genes have been described as highly expressed in the anthocyanin-rich Yan73 “teinturier” cultivar (19). However, the comparative analysis of Yan73 and Cabernet Sauvignon was only able to reveal differences in the expression levels of these genes, as both grape varieties used in this study produce blackberries (19). By contrast, our experimental model addresses the case of red and white calli, with active or inactive anthocyanin pathways, respectively, and is therefore more suitable to uncover the genes that are instrumental to red pigment production.

Overall, the case study shown here with the *MybA1*-correlated genes not only demonstrates the effectiveness of VitExpress in retrieving known genes whose expression is related to pigment metabolism in black grape varieties but it also demonstrates its ability to identify new genes putatively important in anthocyanin biosynthesis and accumulation that may become valuable targets for innovative studies in the future. The new candidate genes provide a robust mean to discriminate between white and black grapes.

### Concluding Thoughts.

There is an undeniable need for a change in viticulture practices to meet present and future environmental requirements while preserving the essential qualities of grape varieties. Obviously, such a transition requires knowledge-based new concepts and the outcome of our present study is in direct line with this objective through the generation of advanced genomic resources on grapevine which provides tools for the investigation of the processes and factors underlying major traits such as tolerance to biotic and abiotic stresses or metabolic pathways defining sensory quality attributes. The generation of a high-quality gap-less telomere-to-telomere genome assembly provides an improved reference genome sequence for grapevine. It represents an essential building block toward the construction of advanced genomics strategies aiming to decipher the mechanisms necessary for the adaptation to environmental changes. The pipeline set up for de novo sequencing and assembly of heterozygous grapevine genomes allowed the unprecedented separation of the two haplo-genomes coexisting in the grape varieties Chasselas and Ugni Blanc. Remarkably, in addition to overcome the obstacle of heterozygosity of grapevine genomes, the haplotype-resolved assemblies open the way to the identification of varieties that share the same haplo-genomes and therefore provide clues into the evolutionary history and genetic relatedness of cultivated grapes. It also paves the way to trace the origins of important traits within landraces and old genotypes that are no longer cultivated through genetic association studies (GWAS) and comparative transcriptomic profiling.

Building on the upgraded assembly and the improved genome annotation based on the integration of de novo single long-read sequencing of full-length RNAs, we developed VitExpress, a web-based expression platform for mining grape transcriptomic data. The open interactive transcriptomic platform provides a genome browser and integrated web tools giving spatiotemporal gene expression patterns, as well as correlation of expression among genes within and across specific organs or conditions. The case study described in the present study shows that VitExpress is not only able to cluster grape into black and white varieties based on the specific expression of anthocyanin metabolism genes, but it also provides new candidate genes potentially important in controlling anthocyanin biosynthesis pathway. Experimental validation of the data delivered by the VitExpress platform clearly sustains the robustness of its versatile tools. The allelic variation within the new candidate genes involved in anthocyanin metabolism may explain the evolutionary mechanisms that separated black and white grapes. Similar studies may apply for the search of genes underpinning tolerance or sensitivity to biotic and abiotic stresses. While so far grapes benefited little from improvement by classical breeding (8) and viticulture is still mainly relying on old varieties obtained hundreds of years ago, there is a need for the development of advanced genomic resources and tools that are instrumental to ensure the transition to a new era where *V. vinifera* could gain new traits to adapt to changing environment. Overall, the resources and tools generated in the study provide means for efficient mining of natural diversity and optimal exploitation of the reservoir of beneficial traits among *V. vinifera* species, and in this regard, it opens the way for the implementation of new breeding technologies aimed to improving grape without losing the varietal initial identity.

## Materials and Methods

### Genome Assemblies.

The assembly combines 50x coverage PacBio HiFi sequencing and Hi-C Sequencing. Contigs were produced using HiFiasm (16). Scaffolds were obtained using Hi-C reads with 3D de novo assembly pipeline (38) and manually reviewed with Juicebox software (39).

### Annotation.

De novo annotation process was led with 30x coverage PacBio IsoSeq combined with evidence from plant protein databanks and 1,600 grape RNA-seq samples. The model was further enriched using the current PNv4 reference model.

### Haplotype Genotyping and Phylogenetic Analysis.

To assess the haplotype-resolved assemblies, a robust subset of 10k SNPs of the 18k SNPs Vitis genotyping array (23) obtained with 783 genotypes was generated. Based on alleles retrieval, phylogenetic trees segregating our genotypes were produced.

### VitExpress Platform and Data Availability.

Raw RNA-seq data were retrieved and filtered from Sequence Read Archive. Only the samples associated with a publication were selected and were manually described. These samples were then processed with a unique RNA-seq quantification pipeline (*SI Appendix*, Fig. S6) and the results stored in a database accessible through the VitExpress Platform (www.vitexpress.gbfwebtools.fr). A publicly accessible website was also created to serve as a central hub for the data generated in the present study (www.grape.resources.gbfwebtools.fr). It also provides tools such as a converter for the different annotation models.

### qPCR Analysis.

Total RNA was extracted from three independent Gamay, Muscat, and Syrah calli using the Plant RNA Purification Reagent (Invitrogen). Following DNase treatment, first-strand complementary DNA was reverse transcribed from 2 mg of total RNA using the Omniscript Reverse Transcription kit (Qiagen). Gene-specific primers (*SI Appendix*, Table S8) were designed by Primer Express software (PE-Applied Biosystems) and checked by BLAST against the grape whole genome. qRT-PCR analyses were performed as described previously (40) to assess the expression of the 7 most highly *MybA1*-correlated genes in the calli of the three different grape varieties. Expression was calculated using the 2-ΔΔCt relative expression method, normalized to ACT, EF1, and UBQ transcript abundance. Each Ct value represents the average of three biological replicates and three technical repeats.

Additional methods implemented in this study are described in detail in the file provided as *SI Appendix*.

## Supplementary Material

Appendix 01 (PDF)

## Data Availability

Genome sequences data have been deposited in www.grape.resources.gbfwebtools.fr (41).
