# Supplementary material for: Haplotype-resolved genome assembly and implementation of VitExpress, an open interactive transcriptomic platform for grapevine
Source: Proc Natl Acad Sci U S A. 2024 May 28;121(23):e2403750121. doi: 10.1073/pnas.2403750121 (PMC11161759; doi:10.1073/pnas.2403750121)
Supplement: Supplementary file 1 — Appendix 01 (PDF) [file pnas.2403750121.sapp.pdf]

PNAS

Supporting Information for

Haplotype-resolved genome assembly and implementation of VitExpress, an open interactive transcriptomic platform for grapevine

Anis Djari[a,1], Guillaume Madignier[a,b,1], Olivia Di Valentin[a], Thibault Gillet[a], Pierre Frasse[a], Amel Djouhri[a], Guojian Hu[a,b], Sebastien Julliard[b], Mingchun Liu[c], Yang Zhang[c], Farid Regad[a], Julien Pirrello[a], Elie Maza[*a], Mondher Bouzayen[*a].

Corresponding authors: Mondher Bouzayen ; Elie Maza

Email: mondher.bouzayen@ensat.fr ; elie.maza@ensat.fr

This PDF file includes:

- Supporting text
- Figures S1 to S7
- Tables S1 to S8
- SI References

Supporting Information Text

Extended methods

DNA Extraction, library construction and sequencing

DNA was isolated from dark treated young leaves using QIAGEN Genomic-tips 100/G kit (Cat No./ID: 10243) following the tissue protocol extraction. Briefly, 0.5g of young leaf material were frozen and ground in liquid nitrogen with mortar and pestle. After 3h of lysis at 50°C and one centrifugation step, the DNA was immobilized on the column. After several washing steps, DNA is eluted from the column, then desalted and concentrated by ethanol precipitation. The DNA is resuspended in EB buffer. DNA quality and quantity were assessed respectively using the Nanodrop-one spectrophotometer (Thermo Scientific, Waltham, MA, USA) and the Qbit 3 Fluorometer using the Qbit dsDNA BR assay (Invitrogen, Thermo Fisher Scientific, Inc). The size of the DNA was assessed using the FemtoPulse system (Agilent, Santa Clara, CA, USA).

On HMW DNA sample, an HIFI SMRTbell® library was constructed using the SMRTbell® Template Prep kit 2.0 (Pacific Biosciences, Menlo Park, CA, USA) according to PacBio recommendations (PN 101-853-100, version 05).

For each genotype, HMW DNA was sheared by using Megaruptor 3 system (Diagenode, Liège Science Park, Belgium) to obtain a 20kb average size. Following an enzymatic treatment on 10µg of sheared DNA sample for removing single-strand overhangs and DNA damage repairs, ligation with overhang adapters to both ends of the targeted double-stranded DNA (dsDNA) molecule was performed to create a closed, single-stranded circular DNA. A nuclease treatment was performed by using SMRTbell® Enzyme Clean-up kit 2.0 (Pacific Biosciences, Menlo Park, CA, USA). A size selection with Blue-Pippin system (Sage Science, Beverly, MA, USA) to remove fragments less than 10kb was done on purified samples with 1X AMPure PB beads (Pacific Biosciences, Menlo Park, CA, USA). The size and concentration of the final library were assessed using the FemtoPulse system (Agilent, Santa Clara, CA, USA) and the Qubit Fluorometer and Qubit dsDNA HS reagents Assay kit (Thermo Fisher Scientific, Waltham, MA, USA), respectively.

Sequencing primer v5 and Sequel® II DNA Polymerase 2.2 were annealed and bound, respectively to the SMRTbell library. The library was loaded on 1 SMRTcell 8M for Chasselas genotype and 2 SMRTcells 8M for Ugni Blanc genotype at an on-plate concentration of 70pM using an adaptive loading. Sequencing was performed on the Sequel® II system with Sequel® II Sequencing kit 2.0, a run movie time of 30 hours with 120 min pre-extension step and Software version 10.1 PacBio by Gentyane Genomic Platform (INRAE Clermont-Ferrand, France).

Chasselas PacBio IsoSeq library preparation

Total RNA was obtained on five grapevine cv. Chasselas tissues (root, stem, leaf, flower and fruit) using the Spectrum Plant Total RNA Kit (Sigma-Aldrich, St. Louis, USA). Analyses of RNA quantity and quality were performed using NanoDrop and Qubit (Thermo Fisher Scientific, Waltham, MA, USA). RNA integrity was also assessed using the Agilent DNF-472 HS RNA (15 nt) kit on the Fragment Analyzer system (Agilent, Santa Clara, CA, USA).

For each vine tissue, 900ng of poly(A) RNA was reverse transcribed into cDNA using the NEBNext® Single Cell/Low Input cDNA Synthesis & Amplification Module (New England Biolabs, Ipswich, Massachusetts, USA) according to PacBio recommendations (PN 101-892-000 Version 01). Amplified cDNA was purified using three different ProNex beads ratio, in order to modulate the full-length cDNA transcript size distribution (0.95X for short transcripts, 0.86X for typical transcripts centered around 2kb and 0.82X for long transcripts enrichment).

The IsoSeq libraries were constructed using SMRTbell® Template Prep kit 2.0 (Pacific Biosciences, Menlo Park, CA, USA) according to PacBio recommendations (PN 101-892-000 Version 01) and each sample was individually barcoded using SMRTbell barcoded adapter plate 3.0 (102-009-200).

The sizes and concentrations of libraries were assessed using the 2100 Bioanalyzer system (Agilent, Santa Clara, CA, USA) and the Qubit dsDNA HS reagents Assay kit. After determining the molarity of each adapter-barcoded sample, they were pooled together according to their size distribution to generate 3 IsoSeq libraries.

Sequencing primer v4 and Sequel® II DNA Polymerase 2.1 were annealed and bound, respectively to the SMRTbell libraries. Each of the three libraries was loaded on 1 SMRTcell 8M at an on-plate concentration of 80pM using adaptive loading. Sequencing was performed on the Sequel® II system with Sequel® II Sequencing kit 2.0, a run movie time of 24 hours with 120 min pre-extension step and Software version 11.0 PacBio by Gentyane Genomic Platform (INRAE Clermont-Ferrand, France).

Hi-C sequencing

The genetic material to produce Hi-C reads was extracted from young leaves of Chasselas and Ugni Blanc genotypes. To ensure the most informative Hi-C contact map we chose an endonuclease-based kit. Thus, the number of fragments produced is increased compared to the kits based on a combination of restriction enzymes.

The Omni-C library was prepared using Dovetail Omni-C™ Kit (Cantata Bio, Scotts Valley, CA, USA) according to the manufacturer's protocol independently for Chasselas and Ugni Blanc. Briefly, starting from 1.5g of young leaves, chromatin was fixed in place in the nucleus. Fixed chromatin was digested with endonuclease DNase I then extracted. Chromatin ends were repaired and ligated to a biotinylated bridge adapter followed by proximity ligation of adapter containing ends. After proximity ligation, crosslinks were reversed and the DNA purified from proteins. Purified DNA was treated to remove biotin that was not internal to ligated fragments. Sequencing library was generated using Illumina-compatible adapters. Biotin-containing fragments were isolated using streptavidin beads before PCR enrichment of the library. The libraries were sequenced on an Illumina NovaSeq6000 platform. Finally, to minimize the sequencing of chimeric reads, an insert size of 390bp and sequencing size of 150bp has been used.

Genomes assemblies

The full assembly process comprises 4 steps. (i) The pre-processing, consisting in HiFi read extraction (subreads correction), (ii) the raw data profiling, (iii) the graph resolution resulting in the draft primary assembly and the haplotype assemblies, and (iv) the Hi-C scaffolding using the contact-map. The produced assemblies were then assessed and polished. In parallel, the organelles' genomes were also assembled using supplementary contigs of hifiasm assembly results coupled with manual reconstruction.

For each HiFi run, only reads considered as viable Circular Consensus Sequences (CCS) were extracted. CCS reads must have at least 3 passes of polymerase and a Phred Score quality greater than 30 (equivalent to read accuracy >0.99). CCS reads were blasted against the NCBI UniVec (1) to detect common contaminants. Only CCS reads displaying less than 10 % match with the contaminant were retained. A 250 bp-long contaminant threshold was applied to exclude nonspecific matches. To check for GC count anomalies of the filtered CCS reads, Kat plot (2) from k-mer matrix table was computed.

Estimated CCS coverage was calculated with a hypothetical 500 Mb *Vitis vinifera* ssp. *vinifera* genome size for both cultivars. K-mer frequencies (21-length) were computed using Jellyfish2. Major metrics such as heterozygosity rate, the percentage of repetitions and estimated genome length were then inferred with GenomeScope 2.0 (3).

HiFi reads were assembled using Hifiasm v.0.16.0-r369 (4) based on its global performance on heterozygous samples and its computational efficiency that allows easy adjustment of parameters. Regarding the heterozygosity of the samples and the use of two batches of CCS reads coming from two different SMRT cells, the similarity threshold for duplicate haplotigs was set up at -s 0.30 after adjustments and imbalance checking between haplotig genomes. Samples with high heterozygosity rate may lead to one assembly being much artificially larger than the other during haplotype phasing process. Therefore, the haplotig deduplication and similarity threshold parameters were set in order to minimize discrepancy in genome length between haplotigs and primary assemblies without altering the overall contiguity.

The Hi-C scaffolding was produced taking advantage of linking information from the Hi-C contact map. Hi-C reads were firstly mapped to the draft genome with juicer (5) with default parameters. Then a first version of the scaffolded assembly was produced with 3D *de novo* assembly pipeline (6). This step was conducted with the parameter -r 0 to prevent automatic polishing, that will be implemented manually in a final step using juicebox software (7). The missing truncated telomeres were then retrieved from the Hi-C free draft assemblies. As for missing telomeres, we aligned independently the chr00 telomeric-like repeat enriched fragments and the raw subreads on telomeres and elongated the chromosomes when subreads aligned perfectly to the informative sequence.

Gap filling of scaffolds junctions

In order to produce the most contiguous assemblies, a remapping of the raw reads against first draft assemblies was processed. Each gap and surrounding areas were manually investigated using Integrative Genome Viewer v.2.12.0 (8). Reads covering the gap with minimum of 5kb overlap on each side with a mapping quality (MAPQ) of at least 60. These reads were then selected to build consensus sequence patches.

Retrieving mitochondrial and chloroplastic genomes
The mitochondrial and chloroplastic sequences were recovered by aligning contigs not related to chromosomes on custom BLAST databases of grapevine organelles' sequences (9). Using a reference-guided assembly strategy, contigs carrying organelle DNA were used to assemble the organelles.

Global synteny. Synteny analysis was handled by genome-to-genome alignment with minimap2 (9). To assess sequence similarity level and overall contiguity, dotplots were produced with DGenies (10). As for syntenic path (co-linear regions and structural rearrangements), SyRI (11) plots were generated.

10k SNPs Vitis genotyping array. A robust subset of 10k SNPs extracted from the 18k SNPs Vitis genotyping array covering 783 genotypes (12) was aligned on each primary and haplotype genome sequences with bwa-mem. Flanking sequences of 5p and 3p were aligned independently and SNP positions were then retrieved according to mapping orientation and CIGAR codes. In this way, only perfectly matching flanking sequences were used. R package "snpReady" (13) was used to evaluate Minor Allele Frequency (MAF), Nei's genetic diversity and Hardy-Weinberg equilibrium statistic for each of the SNP markers. The Euclidean genetic distance was computed on the SNP subset to build the distance matrix. SNPs that did not match the PN12X.v2-based oriented chip were not considered for computing the genetic distance since they most likely correspond to misassembled PN12X.v2 portions of the genome.
In order to produce phylogenetic trees segregation for our genotypes, we conducted a simulation of diploidy for each haplotype. This was achieved by duplicating the alleles, which consequently resulted in them being homozygous for all markers. As for the real heterozygous assemblies we combined allelic information from each haplotype.
Phylogenetic analyses were performed using Discriminant Analysis of Principal Components (DAPC) followed by a K-means clustering with k=8 taking into account the Bayesian Information Criterion (BIC) and using arbitrarily the same cluster number than the 10k SNPs study. The optimal number of PCs was determined by the optim.a.score function from adegenet R v.2.1.10 package (14). DAPC cross-validation was performed with default parameters by checking if the predictive success is maximized and the associated root mean squared error (RMSE) was minimized for our number of PCs.
When associating K-means groups to the most representative geographic region of Vitis genotypes according to Bacilieri et al. (15), each K-means cluster refers to one distinct most representative region as described by Laucou et al. (12).

IsoSeq Model production. The first Step of IsoSeq data processing consists in the production of the full length non chimeric transcripts (FLNC). Circular consensus reads (CCS) were extracted and demultiplexed from raw data. Then the CCS were cleaned from primer sequences and chimeric concatemer. PolyA tails were trimmed and reads were reoriented when needed. The obtained reads were hierarchically clustered to produce one consensus per read cluster. All previous steps were led using PacBio IsoSeq3 (16).
Tissue specific FLNC transcripts were then mapped against Chasselas primary assembly. Using TAMA Collapse from Transcriptome Annotation by Modular Algorithms suite (17), the transcripts aligned were collapsed by tissue. Then all collapsed transcripts were combined into a single transcriptome model using TAMA Merge. To specifically find the protein coding transcripts, the transcriptome model was passed to TAMA ORF to get all open read frames (ORFs) and potential Nonsense-mediated Decays (NMD). The resulting transcripts were then "Blasted" using Blastp against UNIREF. An in-house script was then used to filter Blast results and get the list of best transcript candidates.

***De novo* genome annotation**. Using EugeneEp (18), an automatic gene prediction was produced. The Pipeline was fed with multiple protein and transcriptomic evidences. The predicted annotation was filtered using 1,600 RNA-Seq signal coming from the mapping against the Chasselas genome. In parallel, current PNv4 annotation model was mapped using transcripts and proteins. Only transcripts with at least 80% identity and coverage were kept. The resulting model was then merged with the IsoSeq model using TAMA Merge.
Repetitive elements were identified using several tools. For low complexity regions, we use Dustmasker (NCBI toolkit https://ncbi.github.io/cxx-toolkit/) and for complex repeats, a combination of LTR Harvest (v.2.9.5) (19) and Red (v.2.0) tools (20). Tandem repeats were found using Tandem repeats Finder v.4 (21). To detect *de novo* transposable elements, Repeat modeler (v.2.0) (22) combined with RepeatMasker (v.4) (23) was used.

Genome annotation "lift over". The gene model built from the Chasselas genome assembly was "lifted on" the Ugni Blanc and the four haplo-genomes. During the mapping process, genes from each chromosome were firstly aligned versus the corresponding putative chromosome to minimize biased gene correspondences, given that duplicated genes might catch the best alignment hit. If no solid hit were found, remaining genes were aligned on the other chromosomes. Genes with at least 80% coverage and 80% identity were finally "lifted" to build high quality models. Translocation events between genomes were then investigated by crossing SyRI outputs and liftoff outputs. Screening for transposable elements by RepeatMasker allowed to highlight structural re-arrangements co-localizing with transposable elements.

Annotation models assessment. Assessment was made using BUSCO (24) in transcriptome mode against embryophyta.odb10 and eudicots.odb10. The datasets contain 1,614 universal single-copy genes derived from 50 orthologous genomes and 2,326 universal single-copy genes derived from 30 orthologous genomes, respectively, and allow the evaluation of the gene model completeness.

VitExpress platform production. Data from NCBI SRA databank were filtered and retrieved using NCBI SRA Toolkit v3.0 (25). To ensure the data reliability, only samples associated with a publication were selected. For each selected project a manual description was produced using sample metadata crossed with the information found in the related publication. Then, each sample was processed with a unique RNA-Seq quantification pipeline, using fastQC v0.11.7 (26) for quality check, TrimGalore v0.6.5 (27) for sequence cleaning and trimming, STAR RNA-seq aligner v2.7.9a (28) for the mapping step. Finally, the raw count matrix was produced using FeatureCounts from Subread package v2.03 (29). RNA-seq signal tracks was produced using deepTools v3.0.2 (30). In-house R-script was used to produced normalized expression matrix. The web Application platform was developed using Symfony v.5 (31), a PHP framework coupled with JavaScript libraries: ChartJs (32) for simple charts, Inchlib (33) for HeatMaps, and custom implementation of D3.js (34) for the correlation StatTools. From the RNA-Seq analysis results, an object-relational Database was built using Doctrine (35), a collection of PHP libraries allowing the database storage and object mapping with Symfony.

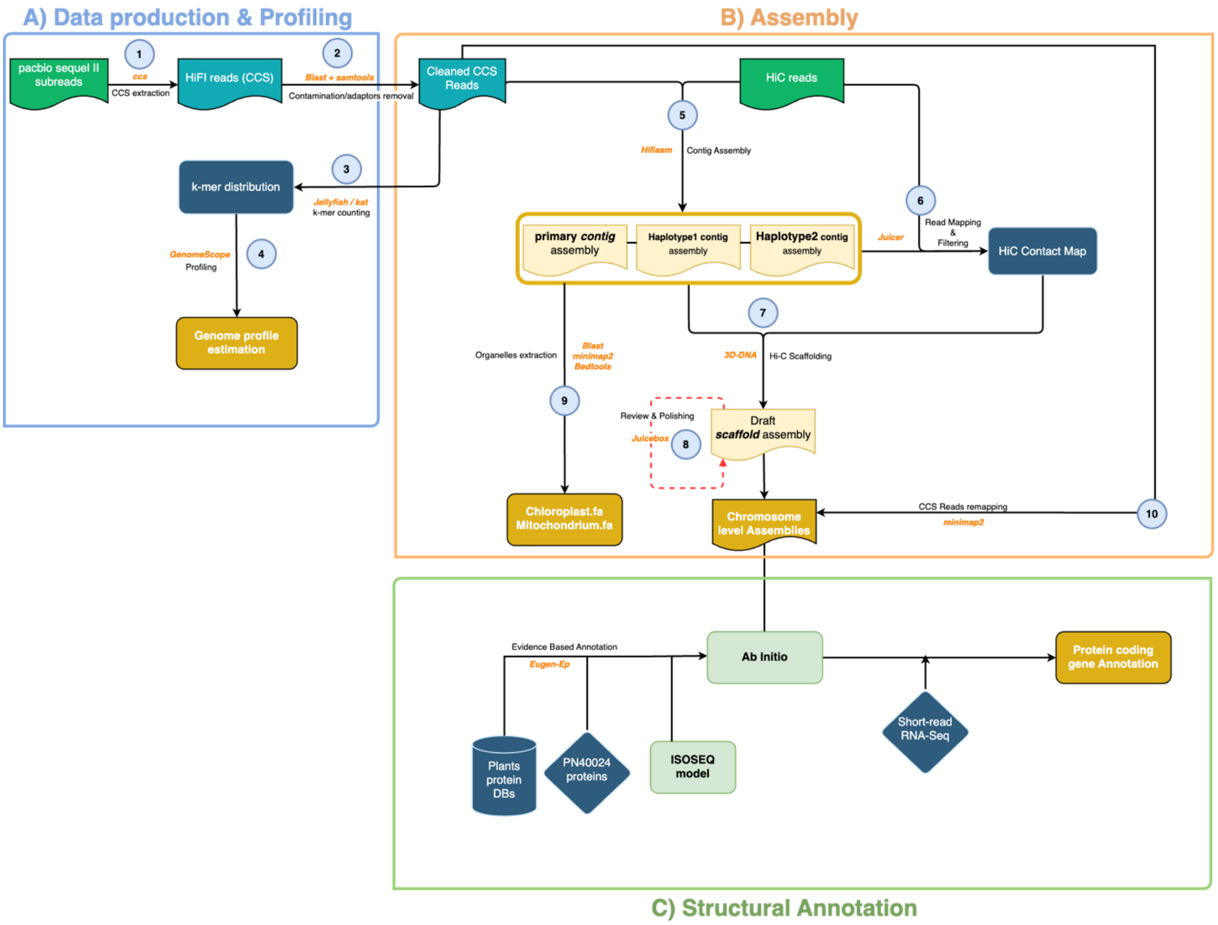

Fig. S1. Full Assembly and annotation pipeline. The workflow of de novo sequencing, assembly and annotation of the grape genome comprises 3 main steps. (A) Raw data production, profiling and validation. (B) Assembly of the circular consensus sequences and integration with the contact map produced by Hi-C. (C) Structural and functional annotation based on Isoseq RNA sequencing data.

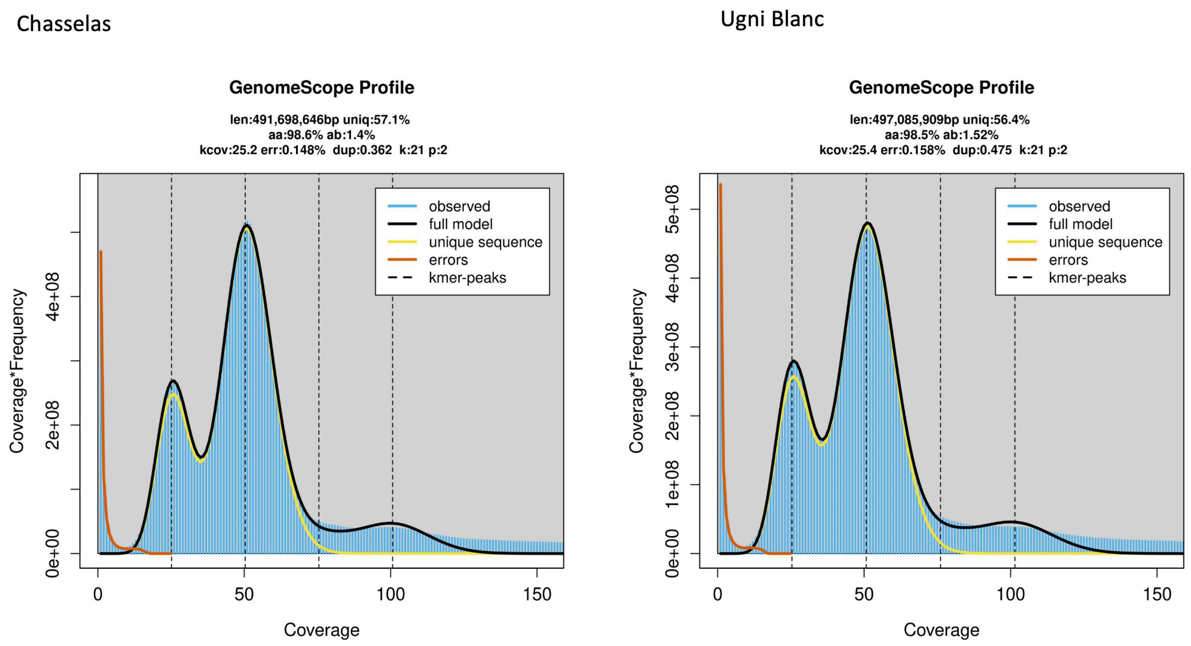

Fig. S2. Raw read Kmer profiling with GenomeScope. First peak stands for unique heterozygous k-mers (25x coverage) and second peak for unique homozygous k-mers (50x coverage). The coverage of 75x and 100x stands for repetitive homozygous and repetitive heterozygous k-mers, respectively. “len” stands for inferred genome length; “uniq” for non repetitive sequence ratio; “aa” for homozygosity rate; “ab” for heterozygosity rate; “kcov” for mean k-mer coverage for heterozygous bases; “err” for error rate of the reads; “dup” for average rate of read duplications; “k” for k-mer size; “p” for ploidy level. Observed (blue): observed k-mer profile; full model (black) : estimated GenomeScope model; unique sequence (yellow): unique k-mer threshold; Errors (red) : error k-mers threshold; k-mer peaks : corresponding to multiple of the kcov peak.

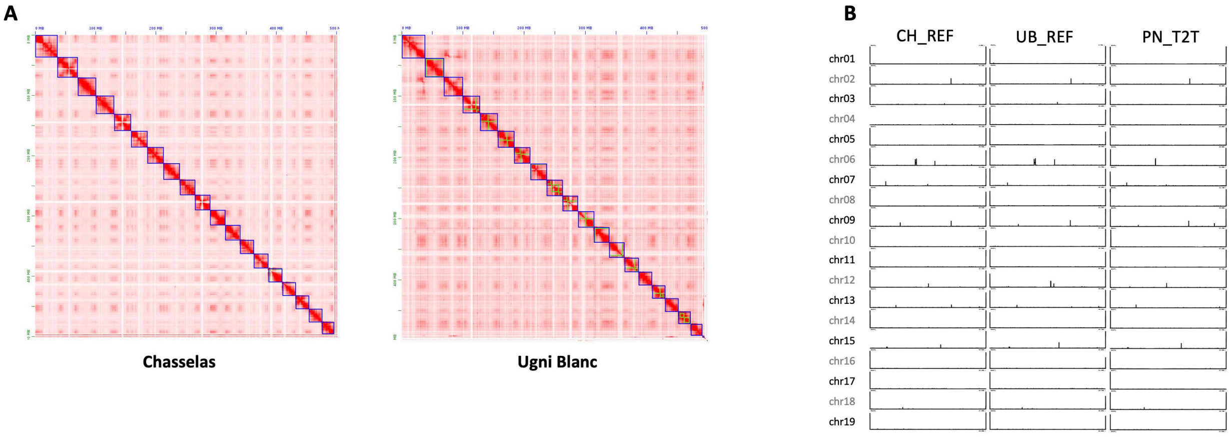

Fig. S3. (A) Hi-C chromosome contact map for Chasselas and Ugni blanc displaying the spatial organization of chromatin. Linkage matrices are built with a 1 kb resolution and loaded on Juicebox. The x-axis represents genomic positions along the genome and the y-axis represents the same positions but mirrored. Each pixel color intensity represents the frequency of interchromosomal and intrachromosomal chromatin contacts between genomic loci. Darker colors indicate higher interaction frequencies. The diagonal line represents self-interactions. (B) Density of the telomeric repeats (AAACCCT)n identified using tidk explore on the chromosomes of Chasselas (left) and Ugni Blanc (middle), and PN_T2T (right). Telomeric motif density is computed with a 1 kb bin size.

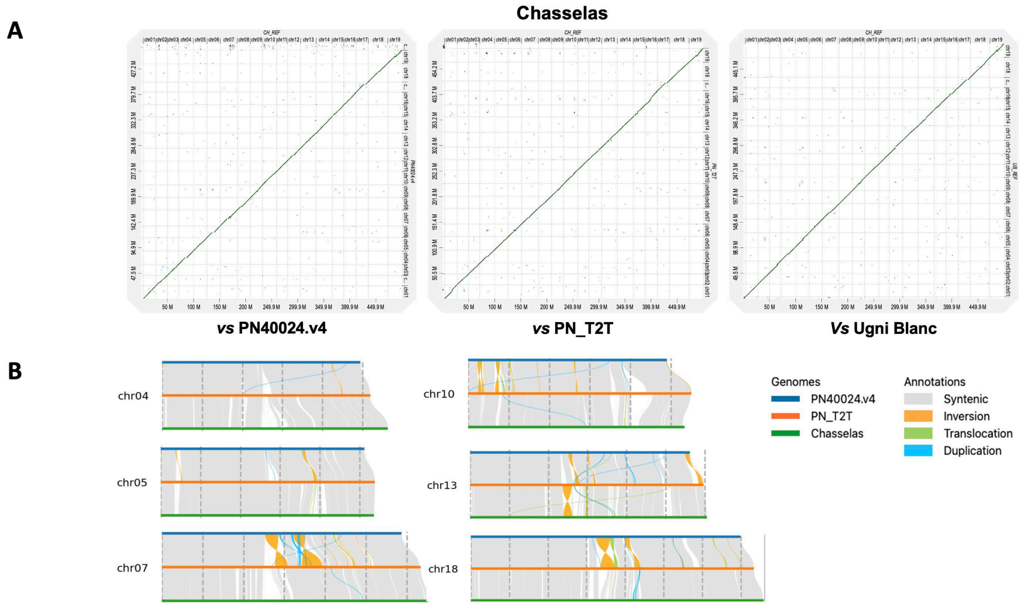

Fig. S4. (A) Dot plots displayed by D-genies showing the collinearity between the genomes of Chasselas vs Pinot Noir (top), Chasselas vs Ugni Blanc (bottom). Grid lines delimit the chromosomal scaffolds. Gaps are depicted by breaks in the global contiguity, horizontal breaks represent gaps in assemblies compared to Chasselas, while vertical breaks indicate gaps in our assembly. (B) Structural variations events (inversions, translocations and duplications) revealed by synteny analysis between PNv4, PN_T2T and Chasselas assemblies.

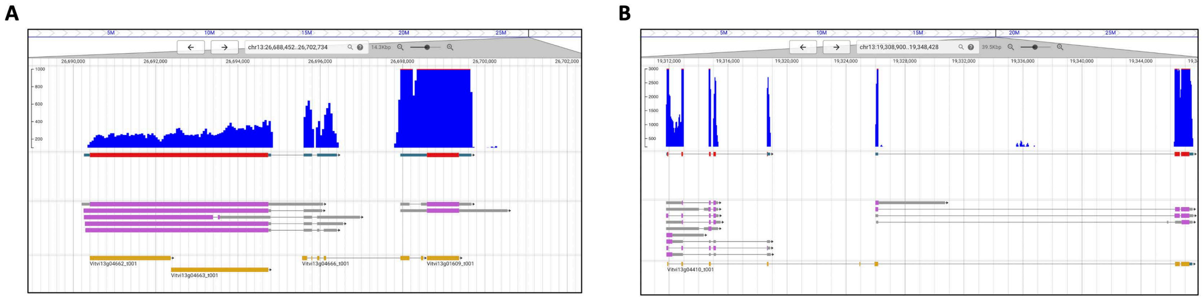

Fig. S5. Examples of reconstruction of PNv4 genes using RNA Isoforms produced by IsoSeq. (A) Fusion event : four PNv4 genes (Vitvi13g04662; Vitvi13g04663; Vitvi13g04666; Vitvi13g01609) defined as only two genes based on IsoSeq. (B) Split event : one PNv4 gene (Vitvi13g04410) shown to correspond to two genes by IsoSeq. First track in blue represents the cumulative RNA-Seq signal from 1,600 samples. Second track in purple correspond to the IsoSeq gene model. Third track represents the PNv4 model remapped against Chasselas assembly.

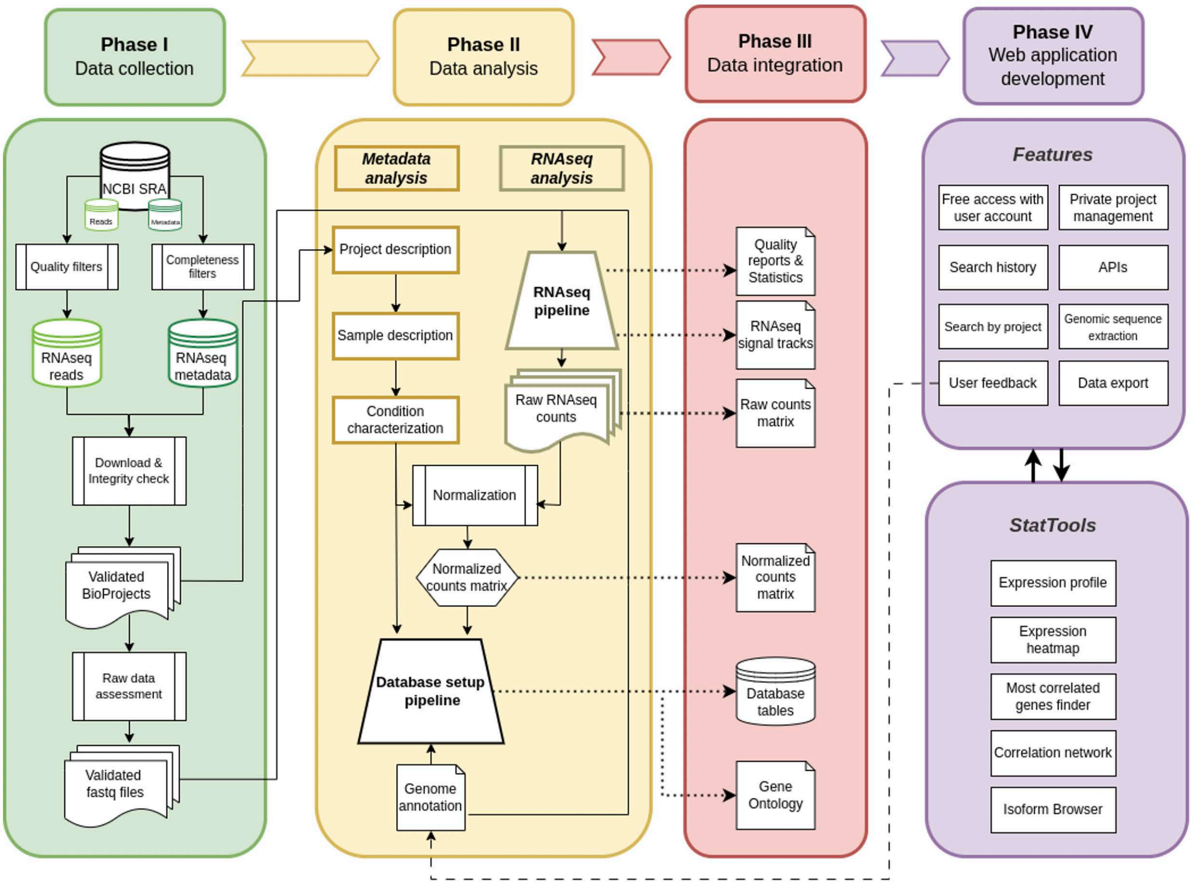

Fig. S6. VitExpress data processing and integration workflow. Phase I : raw data are gathered from NCBI SRA and subsequently evaluated for quality requirement. Phase II : the collected data undergo manual curation and description, followed by RNA-seq quantification. The entire dataset is then normalized. Phase III : the normalized quantification data, along with relevant metadata, are formatted to fit an object relational mapping database and a web application's back-end system. Phase IV: a user-friendly web-based platform is built to facilitate data exploration, analysis and export with statistical and visualization tools.

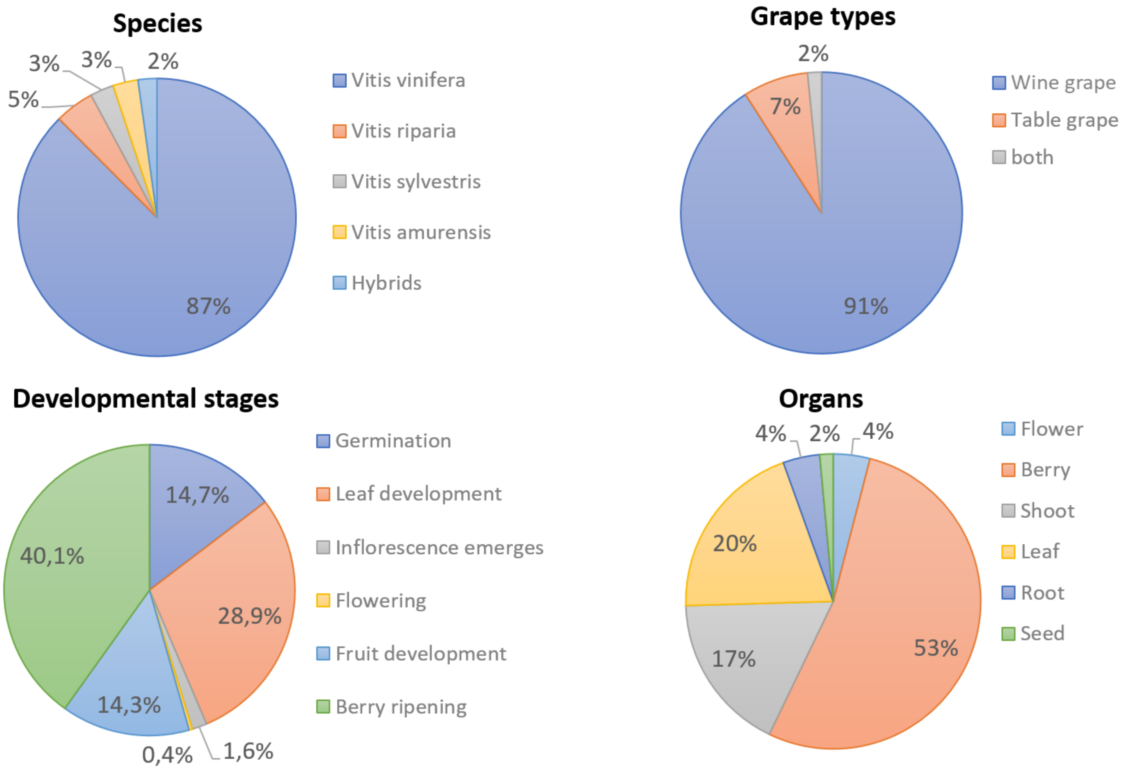

Fig. S7. Data overview of VitExpress database. Pie charts provide the data proportion corresponding to species, grape types, developmental stages and organs. Only manually curated data were included in VitExpress platform.

Table S1. Transposable elements annotation on Chasselas, Ugni Blanc and PN_T2T genome assemblies.

		CH_REF	*UB_REF*	*PN_T2T*
		Number of elements	*Number of elements*	*Number of elements*
**Transposable Elements [1]**		**489,737 (53.11 %)**	**487,202 (52.84 %)**	**486,614 (53.00 %)**
Retroelements		156,490	154,731	154,956
	SINEs	1,649	1,307	1,412
	LINEs	30,491	30,498	30,513
	LTRs	124,350	122,926	123,031
DNA transposons		22,267	22,427	22,418
Unclassified TEs		310,980	310,044	309,240

Table S2. BUSCO transcriptome scores of Chasselas and PN40024.v4 annotation model using Embryophyta.odb10 (1614 genes from 50 orthologous genomes) and Eudicots.odb10 (2326 from 30 orthologous genomes).

	Embryophyta.odb10			***Eudicots.odb10***		
	Chasselas	***PN40024.v4*** [1]	***PN_T2T*** [2]	***Chasselas***	***PN40024.v4*** [1]	***PN_T2T*** [2]
Complete BUSCOs	99.7 % (1,609)	99.0 % (1,598)	98.3 % (1,587)	99.5 % (2,313)	99.0 % (2,301)	98.4 % (2,289)
Complete & single-copy	1,585	1,249	1,243	2,274	1,823	1,821
Complete & duplicated	24	349	344	39	478	468
Fragmented	5	11	22	11	12	20
Missing	0	5	5	2	13	17

[1] *PN40024.v4 annotation version 4.58 from Ensembl Plant released on 2023-11-13*
[2] *PN_T2T annotation model is a lift over of the PN40024.v4 annotation*
Figures between brackets correspond to the total number of complete genes

Table S3. Annotation of protein-coding and non-coding genes of Chasselas and Ugni Blanc haplogenomes.

	CH_REF	*UB_REF*	*CH_HAP1*	*CH_HAP2*	*UB_HAP1*	*UB_HAP2*
Protein-coding genes [1]	32,090	30,937	31,542	31,606	30,804	31,079
Non-coding genes [2]	3,493	3,541	4,558	2,806	2,906	2,894

[1] *number of elements annotated with Blast2GO*
[2] *number of elements screened with tRNAscan-SE, Rnammer and RepeatMasker*

Table S4. Number of genes impacted by structural variants between haplotypes for Chasselas and Ugni Blanc

	Chasselas	Ugni Blanc
Genes impacted by Insertion/deletions events (> 1kb)	793	844
Genes impacted by inversion/translocation events	391	708

Table S5. Tools available in VitExpress.

Visualisation and Statistical Tools	***Description***
Expression profiler	Normalized expression pattern of genes in different chosen conditions
Expression heatmap	Clusterized normalized expression for genes and conditions
Correlation genes finder	Computes and displays the most correlated genes for one selected gene in a chosen conditions
Correlation network	Computes and displays an expression-based, weigthed correlation network for the selected genes
Isoforms Browser	Displays RNAseq signal tracks and gene isoforms on the reference genome for the chosen conditions

Table S6. Ten most correlated genes of *MybA1* identified using the "correlation genes finder" tool in VitExpress

Gene ID	*Correlation*	*Gene Name*	*Annotation*
Vv01g15680	*0.84*	*VvAOMT1*	*Anthocyanin-O-methyltransferase*
Vv16g11110	*0.81*	-	*Antho Multidrug And Toxic compound Extrusion (AnthoMATE)*
Vv08g12510	*0.81*	-	*Solute carrier family 35 member F2*
Vv06g12460	*0.81*	-	*Flavonoid 3',5'-hydroxylase 2-like (F3'5'H)*
Vv16g02100	*0.79*	*UFGT2*	*UDP-glucose:flavonoid 3-O-glucosyltransferase*
Vv04g09160	*0.77*	*GST43*	*Glutathione S-transferase*
Vv01g15670	*0.77*	*AOMT4*	*Flavonoid 3',5'-methyltransferase-like*
Vv02g04950	*0.74*	*LDOX5*	*Leucoanthocyanidin dioxygenase*
Vv03g08520	*0.71*	-	*Serine carboxypeptidase-like 13*
Vv06g03290	*0.70*	-	*Phenylalanine ammonia-lyase*

1 Hugueney et al., 2009; 2 Sparvoli et al., 1994; 3 Conn et al., 2008; 4 Lucker et al., 2010; 5 Sparvoli et al., 1994

Table S7. Putative *Cis*-elements within the promoter regions of the top 7 most highly correlated genes with *MybA1* as predicted by PlantPAN (Chow et al., 2019).

Motif (sequence)	***Vv01g15670***	***Vv01g15680***	***Vv03g08520***	***Vv04g09160***	***Vv06g12460***	***Vv16g02100***	***Vv16g11110***
MBS (CAACTG)	0	1	0	1	2	0	4
MBSI (AaaCSGTTA)	0	0	0	1	0	0	0
MRE (AACCTAA)	0	1	0	4	1	0	2
MYB binding site (CAACAG)	1	1	1	1	1	1	1
MYB binding site (CAACCA)	1	1	0	0	3	2	2
MYB binding site (TAACTG)	0	0	0	0	0	0	0
MYB Hv1 binding site (CCAAT)	3	1	3	2	5	4	3
MYB recognition site (CCGTTG)	0	0	0	0	0	0	0
MYB recognition site (TAACCA)	0	0	4	3	1	1	2

Table S8. Primers used for PCR amplification of MybA1-correlated genes in Gamay, Muscat and Syrah calli.

Gene ID	Forward primer	Reverse Primer
Vv02g11300	CCGAAAAAGCTGCAGGTTGAG	AATCAAGGACCATCTGTTCCCC
Vv01g15680	TGGAAACAAGTGCATACCCAAGAG	GGGACATTCATCAGGCTCCAATAC
Vv16g11110	AGCCAGTGATTTCAGGCGTTG	CCTAGAAGGTAGCCTAGAGGGATTC
Vv08g12510	GTTCAACCTTTCTGCTCTCACATC	AGTATAACCAGTCAACCTTCTGGC
Vv06g12460	GGCGCTCCTCCTGAATTTATTTAC	GAGGATGCTCGGGTTTTTCAAC
Vv16g02100	TAGCACATGAGGCAGTTGGG	GGTCCCCATAAAAGGGCCTG
Vv04g09160	CGATTTCAGGCTGTTTGAGTCG	AACTACCGCTTTCTCCTCCAAG
Vv01g15670	GGAAACAAGTGCATACCCAAGAGAG	AGGGACATTCATCAGGCTCCAATAC

SI References

1. The UniVec Database (2023) (September 11, 2023).

2. D. Mapleson, G. Garcia Accinelli, G. Kettleborough, J. Wright, B. J. Clavijo, KAT: a K-mer analysis toolkit to quality control NGS datasets and genome assemblies. *Bioinformatics* **33**, 574–576 (2017).

3. G. W. Vurture, *et al.*, GenomeScope: fast reference-free genome profiling from short reads. *Bioinformatics* **33**, 2202–2204 (2017).

4. H. Cheng, G. T. Concepcion, X. Feng, H. Zhang, H. Li, Haplotype-resolved de novo assembly using phased assembly graphs with hifiasm. *Nat. Methods* **18**, 170–175 (2021).

5. N. C. Durand, *et al.*, Juicer Provides a One-Click System for Analyzing Loop-Resolution Hi-C Experiments. *Cell Syst.* **3**, 95–98 (2016).

6. O. Dudchenko, *et al.*, De novo assembly of the Aedes aegypti genome using Hi-C yields chromosome-length scaffolds. *Science* **356**, 92–95 (2017).

7. N. C. Durand, *et al.*, Juicebox Provides a Visualization System for Hi-C Contact Maps with Unlimited Zoom. *Cell Syst.* **3**, 99–101 (2016).

8. J. T. Robinson, *et al.*, Integrative genomics viewer. *Nat. Biotechnol.* **29**, 24–26 (2011).

9. H. Li, Minimap2: pairwise alignment for nucleotide sequences. *Bioinformatics* **34**, 3094–3100 (2018).

10. F. Cabanettes, C. Klopp, D-GENIES: dot plot large genomes in an interactive, efficient and simple way. *PeerJ* **6**, e4958 (2018).

11. M. Goel, H. Sun, W.-B. Jiao, K. Schneeberger, SyRI: finding genomic rearrangements and local sequence differences from whole-genome assemblies. *Genome Biol.* **20**, 277 (2019).

12. V. Laucou, *et al.*, Extended diversity analysis of cultivated grapevine Vitis vinifera with 10K genome-wide SNPs. *PloS One* **13**, e0192540 (2018).

13. I. S. C. Granato, *et al.*, snpReady: a tool to assist breeders in genomic analysis. *Mol. Breed.* **38**, 102 (2018).

14. T. Jombart, adegenet: a R package for the multivariate analysis of genetic markers. *Bioinformatics* **24**, 1403–1405 (2008).

15. R. Bacilieri, *et al.*, Genetic structure in cultivated grapevines is linked to geography and human selection. *BMC Plant Biol.* **13**, 25 (2013).

16. PacificBiosciences/IsoSeq, Scalable De Novo Isoform Discovery (2023) (September 11, 2023).

17. R. I. Kuo, *et al.*, Illuminating the dark side of the human transcriptome with long read transcript sequencing. *BMC Genomics* **21**, 751 (2020).

18. E. Sallet, J. Gouzy, T. Schiex, "EuGene: An Automated Integrative Gene Finder for Eukaryotes and Prokaryotes" in *Gene Prediction: Methods and Protocols*, Methods in Molecular Biology., M. Kollmar, Ed. (Springer, 2019), pp. 97–120.

19. D. Ellinghaus, S. Kurtz, U. Willhoeft, LTRharvest, an efficient and flexible software for de novo detection of LTR retrotransposons. *BMC Bioinformatics* **9**, 18 (2008).

20. H. Z. Girgis, Red: an intelligent, rapid, accurate tool for detecting repeats de-novo on the genomic scale. *BMC Bioinformatics* **16**, 227 (2015).

21. G. Benson, Tandem repeats finder: a program to analyze DNA sequences. *Nucleic Acids Res.* **27**, 573–580 (1999).

22. J. M. Flynn, *et al.*, RepeatModeler2 for automated genomic discovery of transposable element families. *Proc. Natl. Acad. Sci.* **117**, 9451–9457 (2020).

23. Smit, AFA, Hubley, R & Green, P., RepeatMasker (2013).

24. M. Manni, M. R. Berkeley, M. Seppey, F. A. Simão, E. M. Zdobnov, BUSCO Update: Novel and Streamlined Workflows along with Broader and Deeper Phylogenetic Coverage for Scoring of Eukaryotic, Prokaryotic, and Viral Genomes. *Mol. Biol. Evol.* **38**, 4647–4654 (2021).

25. The NCBI SRA (Sequence Read Archive) Toolkit (2023) (September 11, 2023).

26. Babraham Bioinformatics - FastQC A Quality Control tool for High Throughput Sequence Data (2023) (September 11, 2023).

27. FelixKrueger/TrimGalore at 0.6.10. *GitHub* (2023) (September 11, 2023).

28. A. Dobin, *et al.*, STAR: ultrafast universal RNA-seq aligner. *Bioinforma. Oxf. Engl.* **29**, 15–21 (2013).

29. Y. Liao, G. K. Smyth, W. Shi, featureCounts: an efficient general purpose program for assigning sequence reads to genomic features. *Bioinforma. Oxf. Engl.* **30**, 923–930 (2014).

30. deepTools2: a next generation web server for deep-sequencing data analysis - PubMed (2023) (September 11, 2023).

31. Symfony, Symfony, High Performance PHP Framework for Web Development (2023) (September 11, 2023).

32. Chart.js, A simple yet flexible JavaScript charting library for the modern web (2023) (September 11, 2023).

33. C. Škuta, P. Bartůněk, D. Svozil, InCHlib – interactive cluster heatmap for web applications. *J. Cheminformatics* **6**, 44 (2014).

34. D3 by Observable | The JavaScript library for bespoke data visualization (2023) (September 11, 2023).

35. Doctrine: PHP Open Source Project (2023) (September 11, 2023).
